# Rapid Isolation of *Staphylococcus aureus* Pathogens from Infected Clinical Samples Using Magnetic Beads Coated with Fc-Mannose Binding Lectin

**DOI:** 10.1371/journal.pone.0156287

**Published:** 2016-06-08

**Authors:** A. Bicart-See, M. Rottman, M. Cartwright, B. Seiler, N. Gamini, M. Rodas, M. Penary, G. Giordano, E. Oswald, M. Super, D. E. Ingber

**Affiliations:** 1 Wyss Institute for Biologically Inspired Engineering, Harvard University, Boston, Massachusetts, United States of America; 2 Hospital Joseph-Ducuing, Toulouse, France; 3 Universite de Versailles, St Quentin, France; 4 INSERM 1043, Toulouse, France; 5 Harvard Medical School and Boston Children’s Hospital, Boston, Massachusetts, United States of America; 6 Harvard John A. Paulson School of Engineering and Applied Sciences, Cambridge, Massachusetts, United States of America; University of Leicester, UNITED KINGDOM

## Abstract

Here we describe how *Staphylococcus aureus* bacteria can be rapidly isolated from clinical samples of articular fluid and synovial tissue using magnetic beads coated with the engineered chimeric human opsonin protein, Fc-mannose-binding lectin (FcMBL). The FcMBL-beads were used to capture and magnetically remove bacteria from purified cultures of 12 *S*. *aureus* strains, and from 8 articular fluid samples and 4 synovial tissue samples collected from patients with osteoarthritis or periprosthetic infections previously documented by positive *S*. *aureus* cultures. While the capture efficiency was high (85%) with purified *S*. *aureus* strains grown *in vitro*, direct FcMBL-bead capture from the clinical samples was initially disappointing (< 5% efficiency). Further analysis revealed that inhibition of FcMBL binding was due to coating of the bacteria by immunoglobulins and immune cells that masked FcMBL binding sites, and to the high viscosity of these complex biological samples. Importantly, capture of pathogens using the FcMBL-beads was increased to 76% efficiency by pretreating clinical specimens with hypotonic washes, hyaluronidase and a protease cocktail. Using this approach, *S*. *aureus* bacteria could be isolated from infected osteoarthritic tissues within 2 hours after sample collection. This FcMBL-enabled magnetic method for rapid capture and concentration of pathogens from clinical samples could be integrated upstream of current processes used in clinical microbiology laboratories to identify pathogens and perform antibiotic sensitivity testing when bacterial culture is not possible or before colonies can be detected.

## Introduction

Diagnosis of blood infections and infections of complex tissues, such as articular joints, commonly rely upon the use of bacterial cultures to isolate and identify the infectious microorganisms. However, most patients have negative blood cultures, and even when the infectious microbe does grow in vitro, it can still take one to many days before the pathogen is identified [1]. The development of more rapid infection diagnostics has been hindered by the lack of methods to extract bacteria directly from complex biological samples, which would greatly shorten the time required to initiate cultures and document the cause of infection. Several approaches, such as charge-based separation or immunomagnetic capture using specific antibodies, have been explored to achieve bacterial purification [2, 3]. *Staphylococcus aureus*, which is one of the most common causes of osteoarthritis infections [4], also has been captured using immunoglobulins that bind to protein A found on these surface of these bacteria [5]. However, none of these methods work in complex biological fluids, such as those found in clinical tissue samples.

Mannose binding lectin (MBL) is a key opsonin component of the lectin pathway associated with innate immunity, which activates the complement cascade [6, 7]. In the presence of pathogens, MBL binds carbohydrates on the surface of microbial cells in a calcium-dependent manner, targeting complement activation and opsonizing the pathogens for phagocytosis [8]. MBL has an extremely broad-spectrum opsonin activity in that it binds carbohydrates found on the surfaces of a wide range of pathogens including more than 90 different Gram positive and negative bacteria, viruses, fungi and parasites [9–11]. In past work focused on the development of a method to cleanse septic blood without knowing the identity of the infectious agent, we genetically engineered a new version of this human opsonin by fusing the carbohydrate recognition region of MBL to the flexible neck of the Fc portion of IgG1 [12, 13]. These studies confirmed that FcMBL is similarly capable of binding a wide range of pathogens, including those commonly involved in osteoarticular infections, such as *S*. *aureus* [12].

In the present study, we developed a rapid method for isolating *S*. *aureus* bacteria from clinical samples of musculoskeletal tissues, including joint fluids and periprosthetic tissues, by leveraging the generic opsonin capability of FcMBL. This technique was tested in osteoarticular infections because diagnosis can be challenging and require longer cultures, especially in chronic cases and in orthopedic implants where bacterial load is low, and the pathogens often grow as part of a biofilm [14]. While molecular diagnostic approaches can be used to identify *S*. *aureus* and determine whether it is resistant to methicillin, they can not be used to carry out full antibiotic susceptibility testing (AST), which again requires isolation and culture of living bacteria [15]. Thus, development of a method that can isolate sufficient numbers of bacterial cells directly from clinical samples to perform PCR, MALDI-TOF and phenotypic AST without requiring extended culture could significantly advance clinical practice.

## Materials and Methods

### Clinical samples

Articular fluids and musculoskeletal tissues were collected from potentially infected body sites of patients suspected of osteoarthritis or periprosthetic infection. These samples were taking in the course of standard care and the purpose of their use was initially for additional analysis, if necessary. According to French regulation, this type of study is IRB exempt and does not require patient informed consent. We used an extensive biobank of joint samples from cases of implant infections with complete bacteriological and clinical documentation. The samples (waste samples) used in this study originated from replicate samples prospectively taken to allow a retrospective detailed analysis using molecular or other techniques. Four weeks after the surgery, the samples were no longer required for clinical use and they remained stored at -20°C for at least 6 months prior to use in this study; all samples and data were de-identified. For experimental procedures, each sample was thawed and adjusted to a minimum volume of 1 ml with 0.9% NaCl. The solid tissues were disrupted using bead milling for 2.5 minutes, as previously described [16], and 50 μl of the milled suspension was plated on blood agar to confirm viability and quantify bacterial load.

### Opsonin engineering and bead production

**Fig 1:**
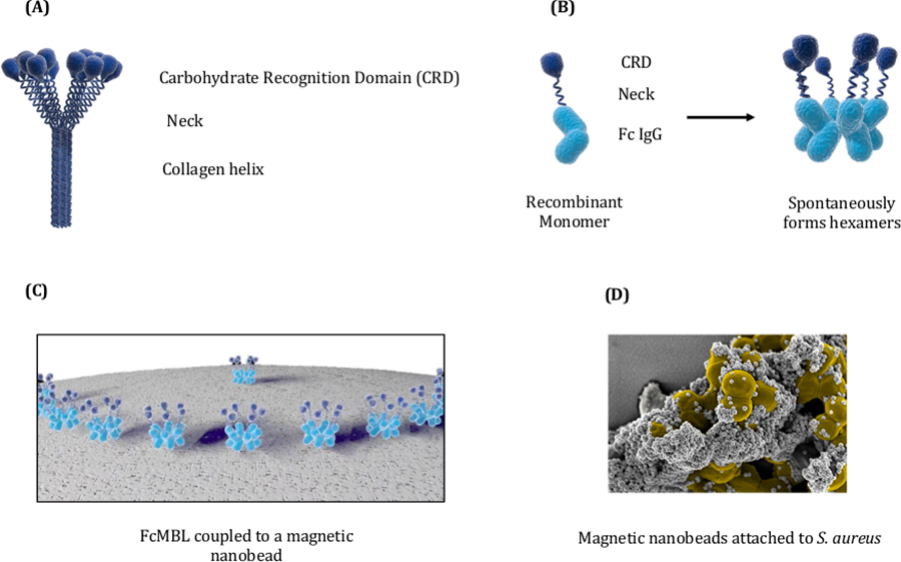
Engineering of Fc-Mannose Binding Lectin (FcMBL). (A) Engineering of FcMBL is initiated by removing the collagen helix domain from native MBL. (B) The Neck and CRD domains of MBL are fused to the Fc region of a human IgG1. (C) N-terminal aminooxy-biotinylation allows its oriented attachment to magnetic nanobeads coated with streptavidin. (D) Electron micrograph showing *S*. *aureus* coated with multiple bound 128 nm FcMBL-beads.

The engineering and biotinylation of FcMBL has been published previously [12]. Briefly, FcMBL (accession code KJ710775() is a fusion protein consisting of the MBL carbohydrate recognition domain and neck region fused to the Fc region of human IgG1. FcMBL is biotinylated at its N-terminus to permit oriented attachment to streptavidin-coated magnetic beads (Fig 1). Twenty-five μg of N-terminally biotinylated FcMBL protein were incubated with 1 mg of beads (approximately 10^10^ superparamagnetic nanobeads, 500 nm diameter; Ademtech, France), which corresponds to approximately 36,000 FcMBL molecules per bead. These beads were then incubated with 4.7 μg of biotinylated polyethylene glycol (PEG)-1K (Nanocs Inc) to block unbound streptavidin sites. Fc beads were generated using the same method, but biotinylated human Fc was substituted for biotinylated FcMBL. Control beads were generated using the same protocol, but biotinylated FcMBL was omitted in the incubation step. Unbound protein and PEG were removed from FcMBL, Fc and control beads by washing the beads three times in phosphate buffered saline (PBS) with 1% bovine serum albumin (BSA) and the beads were then stored in PBS supplemented with 1% BSA and 10 mM EDTA at a bead concentration of 5 mg/ml.

### Pathogen capture assay

The ability of FcMBL-coated beads to capture bacterial strains was evaluated by adding 10 μl of beads coated with either FcMBL or PEG-biotin (non-specific binding control) or Fc (Protein A–Fc binding control) to a 1 ml sample containing 100 μl of a bacterial solution (10^4^ CFU/ml) and 900 μl of Tris 50 mM, NaCl 150 mM, Tween 0.05% supplemented with 5 mM calcium (TBS-T Ca^2+^). Beads were incubated for 20 min at room temperature with gentle agitation, removed from solution using a magnet separator (K & J Magnetics, Inc, PA), and then both the supernatants and beads were plated on culture dishes for quantification of pathogen capture. The capture efficiencies were determined as the percent reduction in the number of bacteria in the FcMBL supernatant or Fc supernatant compared to the PEG-biotin supernatant. Number of bacteria adhering to FcMBL, Fc, and control beads were also determined by counting plated colonies. Capture efficiency was not further improved by increasing the amount of beads used. FcMBL capture efficiencies from infected joint samples were determined using the same procedure described above.

### Analysis of immunoglobulin inhibition of FcMBL capture

To determine inhibition of FcMBL capture of *S*. *aureus* by endogenous immunoglobulins,10 μl of 1 mg/ml human total Ig (Jackson Immuno Research, PA) was added to 100 μl of a solution containing a clinical *S*. *aureus* strain (10^4^ CFU/ml) in PBS. The bacteria/IgG solution was incubated for 30 minutes at room temperature with gentle agitation and washed twice before carrying out the FcMBL-bead capture assay.

Removal of endogenous immunoglobulin was used to determine the inhibition of FcMBL capture of *S*. *aureus* by immunoglobulins present in the infected joint samples. Endogenous immunoglobulins were depleted using Protein A coated superparamagnetic beads (Thermo scientific, MA). Ten μl (100 μg) of protein A-coated beads were added to 100 μl synovial fluid and incubated for 30 min at room temperature. Protein A beads were separated from the sample using a magnetic separator. FcMBL capture of *S*. *aureus* in the immunoglobulin-depleted supernatant was determined using the pathogen capture assay.

### Clinical sample preparation

Bead-milled tissue or articular fluid samples (500 μl) were added to 1 ml of sterile water, incubated for 10 min, and centrifuged (10 min at 9,000 rcf). The pellets were then either analyzed directly by resuspension in TBS-T 5 mM Ca^2+^ followed by FcMBL bead capture assay or resuspended in 30 μl hyaluronidase (working concentration of 300 μg/ml; Sigma-Aldrich, Inc, MO. Product No. H 4272), 300 μl mix of proteases including chymotrypsin, trypsin, carboxypeptidase, amino peptidase, phosphatases (generating an optimal working concentration of proteases of 4 mg/ml; Roche, Switzerland. Product No. 10 165 921 001), and 1 ml phosphate buffered saline (PBS) at pH 6). The enzyme solution was agitated for 60 min at 37°C, centrifuged again, and then the pellet was resuspended with 1 ml protease inhibitor (Roche, Switzerland) for 30 min at room temperature, centrifuged, and resuspended in 1 ml TBS-T Ca^2+^ for the pathogen capture assay.

### Downstream clinical assays

Molecular detection and quantitation of *S*. *aureus* was performed using CE-IVD marked Genexpert SA/MRSA SSTI Gen3 assay on a GX IV real time polymerase chain reaction (RT-PCR) instrument (Cepheid, Marens Scopont, France) according to the manufacturer’s recommendations. Briefly, 100 μl of a sample or bead suspension were added to the sample suspension vial, vortexed, and the suspension transferred to the sample port of the test cartridge. The relative number of *S*. *aureus* genome copies is extrapolated from the CT of the *spa* gene reported by the instrument.

MALDI-TOF identification was performed on a Bruker Microflex LT instrument and the Biotyper V3 software following formic acid extraction performed on the target. Bacteria outgrown in Tryptic Soy Broth from beads-bound *S*. *aureus* were centrifuged and the pellet resuspended in 2 μl acetonitrile (Sigma, France) and spotted on a stainless steel target and dried under a fume hood. Two μl of MALDI matrix (saturated solution of α-cyano-4-hydroxycinnamic acid [HCCA; Bruker Daltonics] in 50% acetonitrile and 2.5% trifluoroacetic acid) were then applied to the spot and dried. Two μl of formic acid were then added to enhance protein extraction. The plate was then analyzed using the Biotyper software.

### Statistical analysis

All datasets were analyzed using Student’s unpaired two-tailed t-test as implemented in Prism 6.0g for OSX. A p-value < 0.05 was used as a threshold for statistical significance.

## Results

**Fig 2:**
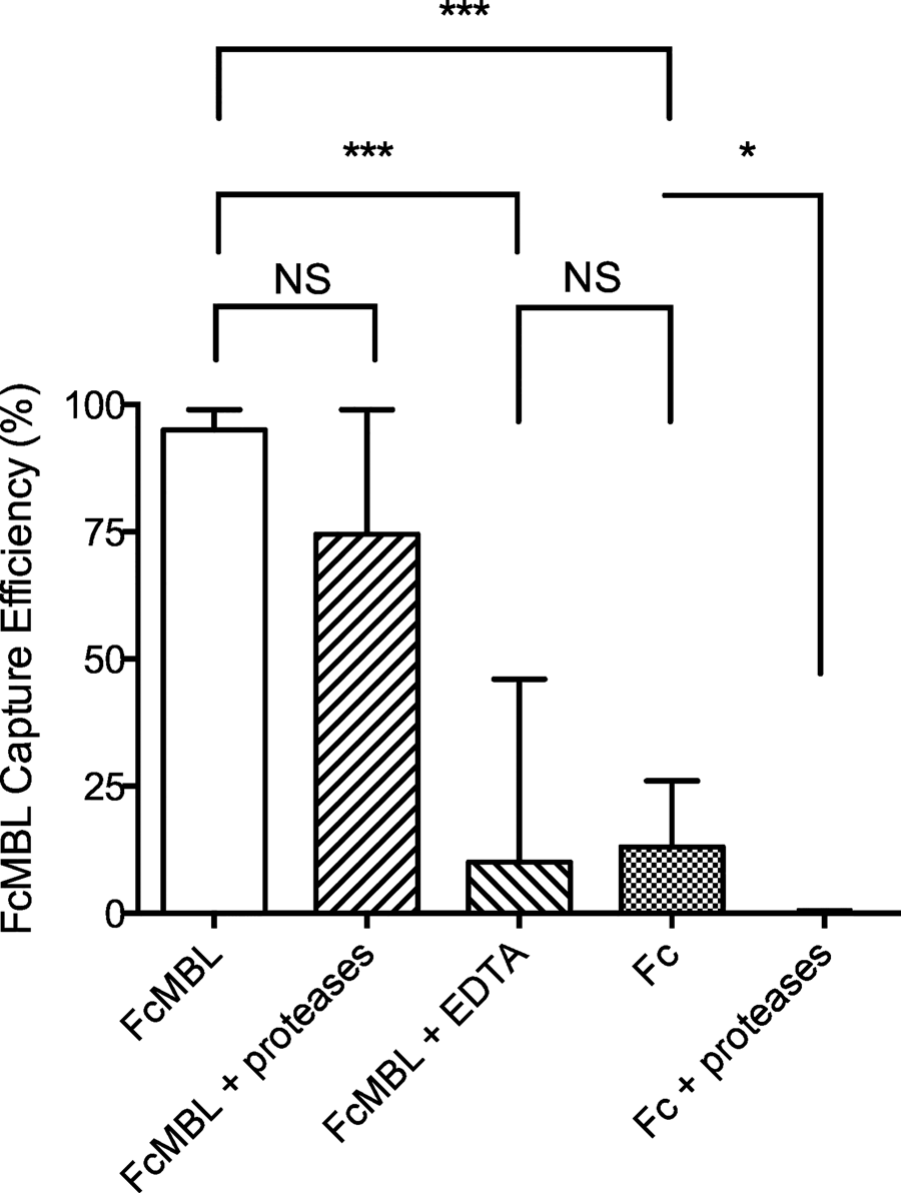
Pathogen capture efficiencies using magnetic separations for 12 *S*. *aureus* clinical isolates grown *in vitro*. All cultured bacterial strains isolated isolated from infected tissues bound to FcMBL-beads (FcMBL) and produced capture efficiencies ranging from 30 to 100% (mean: 84%). Use of Fc-coated magnetic beads (Fc) or FcMBL-beads in the presence of EDTA to support only binding to surface protein A resulted in much more heterogeneous response, with capture frequencies from 0 to 80% (mean Fc: 21%, mean EDTA: 24%). Fc capture was totally abolished by treatment with proteases that can degrade surface protein A (Fc + proteases), while FcMBL capture was conserved (mean: 68%) under the same conditions (FcMBL + proteases). ***: p-value < 0.0001; *: p-value < 0.05; NS: not significant

Initial capture assays using ATCC reference strains of *S*. *aureus* revealed that 80 to 100% of the bacteria could be bound using FcMBL beads. Encouraged by the high binding efficiency of these reference strains, we tested clinical samples of 8 articular fluids and 4 musculoskeletal tissues that had previously been found to be infected with *S*. *aureus*, as well as cultured strains isolated from these samples. The origin of the samples is summarized in Table 1. However, when we applied the FcMBL capture directly to these tissues, the results were disappointing with capture rates < 5%, even though the tissues underwent bead-milling to release the pathogens. But when we carried out studies with cultured pathogens that were isolated from these same tissue specimens, we found that the FcMBL beads captured these clinical strains of *S*. *aureus* with a very high (> 90%) efficiency (Fig 2). FcMBL could bind to *S*. *aureus* through two distinct high affinity mechanisms: the carbohydrate recognition domain of MBL binds specifically to cell wall techoic acid (WTA) [17] and its Fc domain, which also binds to *S*. *aureus* protein A [18]. When we carried out similar studies using beads coated with Fc alone, or with FcMBL beads in the presence of EDTA (to promote only protein A binding while preventing carbohydrate binding), we observed reduced binding and greater heterogeneity, confirming the importance of the FcMBL’s carbohydrate recognition domain for efficient pathogen capture (Fig 2).

**Table 1:** Clinical Tissue Samples Infected with *S*. *aureus*.

N°	Joint	Presence of Implant.	Tissue	Infection^a^	Suscept^b^	Direct ex^c^	Growth^d^	Load^e^
1	knee	yes	Syn. fl	Acute	MSSA	Pos	na	1.7.10^4^
2	knee	no	Syn. fl	Acute	MSSA	Pos	48H	8.10^2^
3	ankle	no	Syn. fl	Acute	MSSA	Pos	24H	1.9.10^5^
4	knee	yes	Syn. fl	Acute	MSSA	Neg	24H	6.5.10^4^
5	hip	yes	Syn. fl	Chronic	MSSA	na	24H	1.10^5^
6	hip	yes	Syn. fl	Acute	MSSA	Pos	na	6.10^2^
7	shoulder	yes	Syn. fl	Acute	MSSA	Neg	48H	1.1.10^4^
8	hip	yes	Syn. fl	Acute	MSSA	na	24H	3.5.10^5^
9	knee	no	Tissue	Acute	MRSA	Pos	24H	1.10^5^
10	knee	yes	Tissue	Acute	MSSA	na	na	3.6.10^4^
11	knee	yes	Tissue	Chronic	MSSA	Pos	48H	1.10^6^
12	hip	yes	Tissue	Chronic	MSSA	Neg	48H	2.2.10^3^

^a^Acute and chronic infection: clinical signs for less or more than 3 months.

^b^Suscept.: MRSA: Meticillin Resistant *S*. *aureus*; MSSA: Meticillin Susceptible *S*. *aureus*.

^c^Direct ex: 50 μl of sample were smeared on a microscope slide and Gram stained. Pos: observation of clusters of Gram positive cocci; Neg: absence of visible bacteria.

^d^Time required to observe colonies upon initial culture

^e^Load: *S*. *aureus* enumeration (in CFU/ml) determined by plating serial dilutions of the sample.

**Fig 3:**
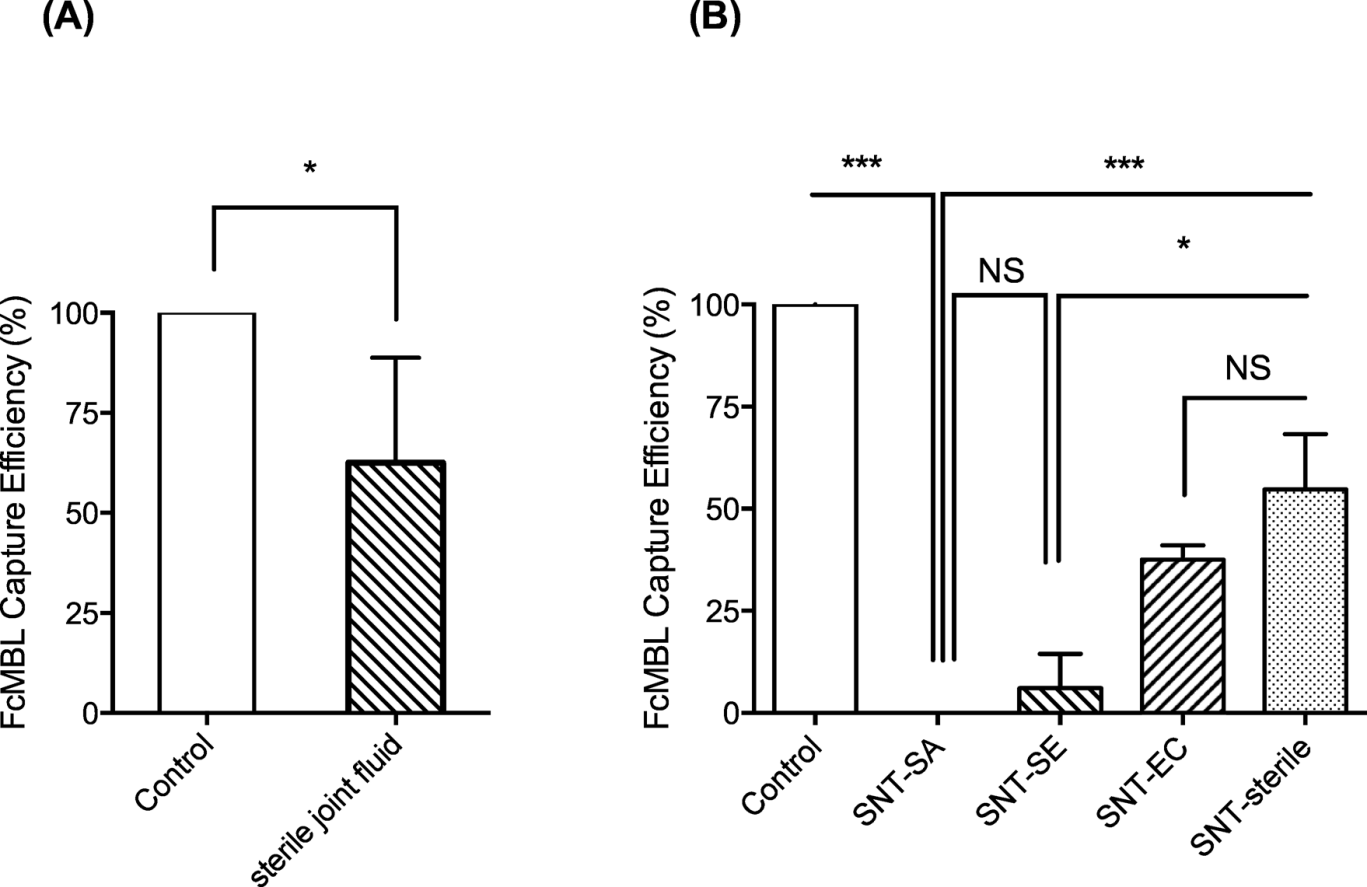
Inhibition of pathogen capture using FcMBL by soluble components in tissue samples. (A) Capture of cultured *S*. *aureus* strains isolated from clinical samples using FcMBL-beads (Control) was partially inhibited by incubation with sterile joint fluids (mean: 62%). (B) Capture was totally inhibited by centrifuging articular fluids infected with *S*. *aureus* and adding the supernatant (SNT-SA). Partial inhibition was produced using the SNT from articular fluid infected with *S*. *epidermidis* (SNT-SE, mean: 6%), *E*. *coli* (SNT-EC, mean: 37%) or sterile articular fluid from non infected patients (SNT-sterile, mean: 55%). ***: p-value < 0.0001; *: p-value < 0.05; NS: not significant

We then explored why the capture method did not work in more complex joint fluid or tissue samples. We assumed that inhibition would be due to factors present in samples, which interact with FcMBL. First, we pre-incubated FcMBL beads with a sterile sample of joint fluid and then evaluated the ability of these beads to bind a cultured *S*. *aureus* strain. These studies revealed that pre-incubation in this complex biological fluid significantly reduced binding between by 40 to 100% (p = 0.0291; Fig 3A). We then centrifuged the sterile joint fluid sample, collected the soluble supernatant, pre-incubated the beads, and repeated the binding study with the cultured bacteria, and found that this treatment inhibited binding of *S*. aureus in the same way. More inhibition was observed using supernatants from filter-sterilized articular fluids isolated from patients infected with *E*. *coli* and *S*. *epidermidis*, *a*nd binding was completely inhibited using supernatants from joint fluids infected with *S*. *aureus* (Fig 3B). Taken together, these observations suggested that soluble components in joint fluid inhibit FcMBL-bead binding to *S*. *aureus* by physically blocking its ability to adhere to the bacterial cell surface.

**Fig 4:**
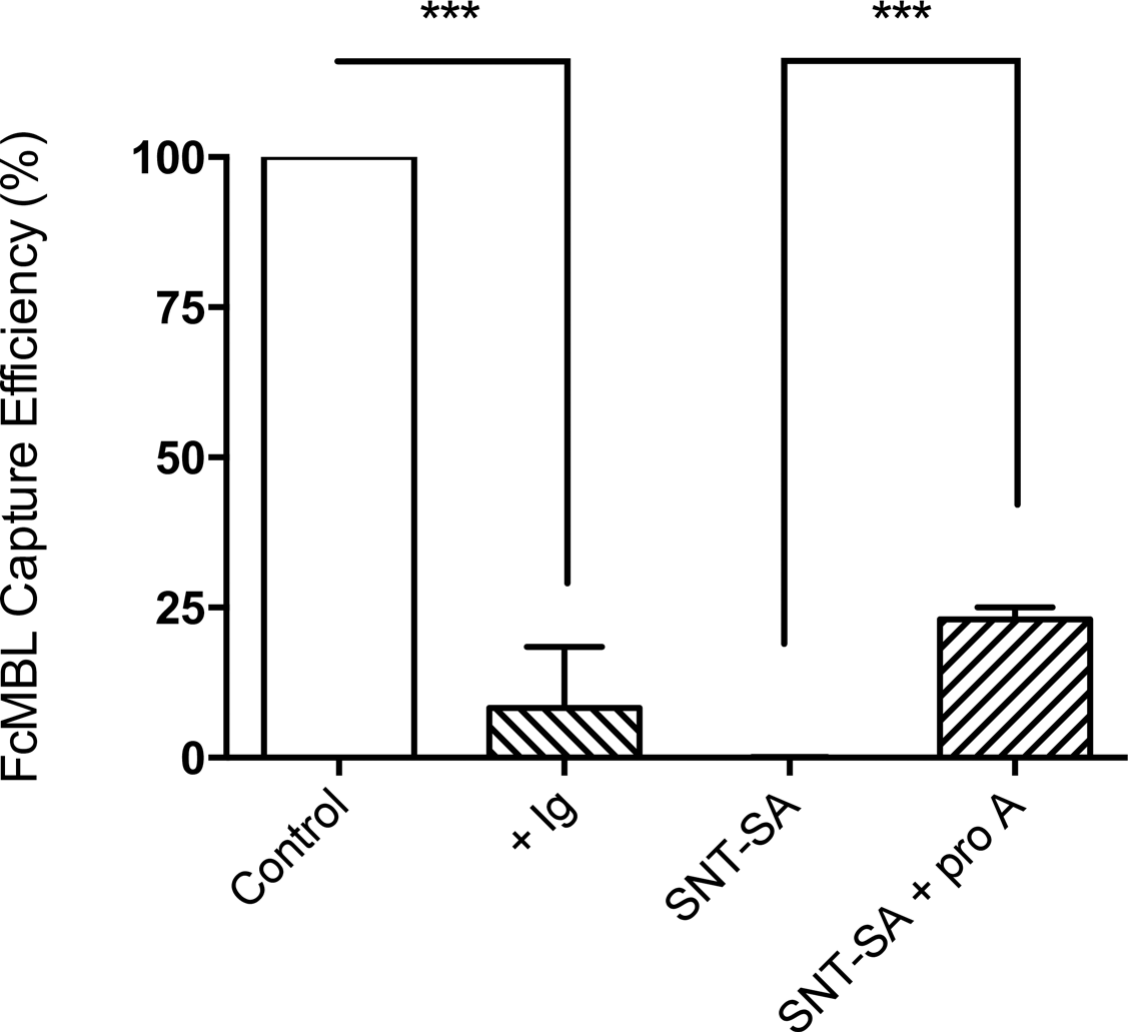
Inhibition of *S*. *aureus* capture by human immunoglobulin (Ig). Capture of cultured *S*. *aureus* strains isolated from clinical samples using FcMBL-beads (Control) was inhibited by adding 1 mg/ml total human Ig (+ Ig, mean: 8%). Depleting Ig in the supernatant of centrifuged clinical samples infected with *S*. *aureus* with protein A-coated magnetic beads partially restored the capture (SNT-SA + ProA, man: 23%). ***: p-value < 0.0001.

To explore the possibility that Ig proteins might contribute to this inhibition, we carried out the FcMBL-bead capture assay in the presence of human total Ig, and found that it similarly inhibited bead binding to cultured *S*. *aureus* (Fig 4). Importantly, depleting *S*. *aureus* infected joint fluids of endogenous Ig using protein A-coated magnetic beads partially restored the ability of the FcMBL beads to capture cultured pathogens in the same joint fluids (Fig 4).

To minimize interference by these soluble inhibitory factors, we performed FcMBL-bead capture using resuspended solutions of the washed pellets from the clinical samples; however, the capture rates only improved marginally (from < 5% to 40%). One possibility is that IgG bound to the surfaces of the pelleted bacteria physically masked the carbohydrate binding sites for FcMBL. Thus, we added an enzymatic digestion step using a mixture of proteases (chymotrypsin, trypsin, carboxypeptidase, amino peptidase, and phosphatases at a 4 mg/ml final concentration) to remove any proteins. This significantly improved pathogen capture from articular fluids, but results were still variable, with heterogeneous binding efficiencies ranging from 40 to 100% (mean: 68%).

**Fig 5:**
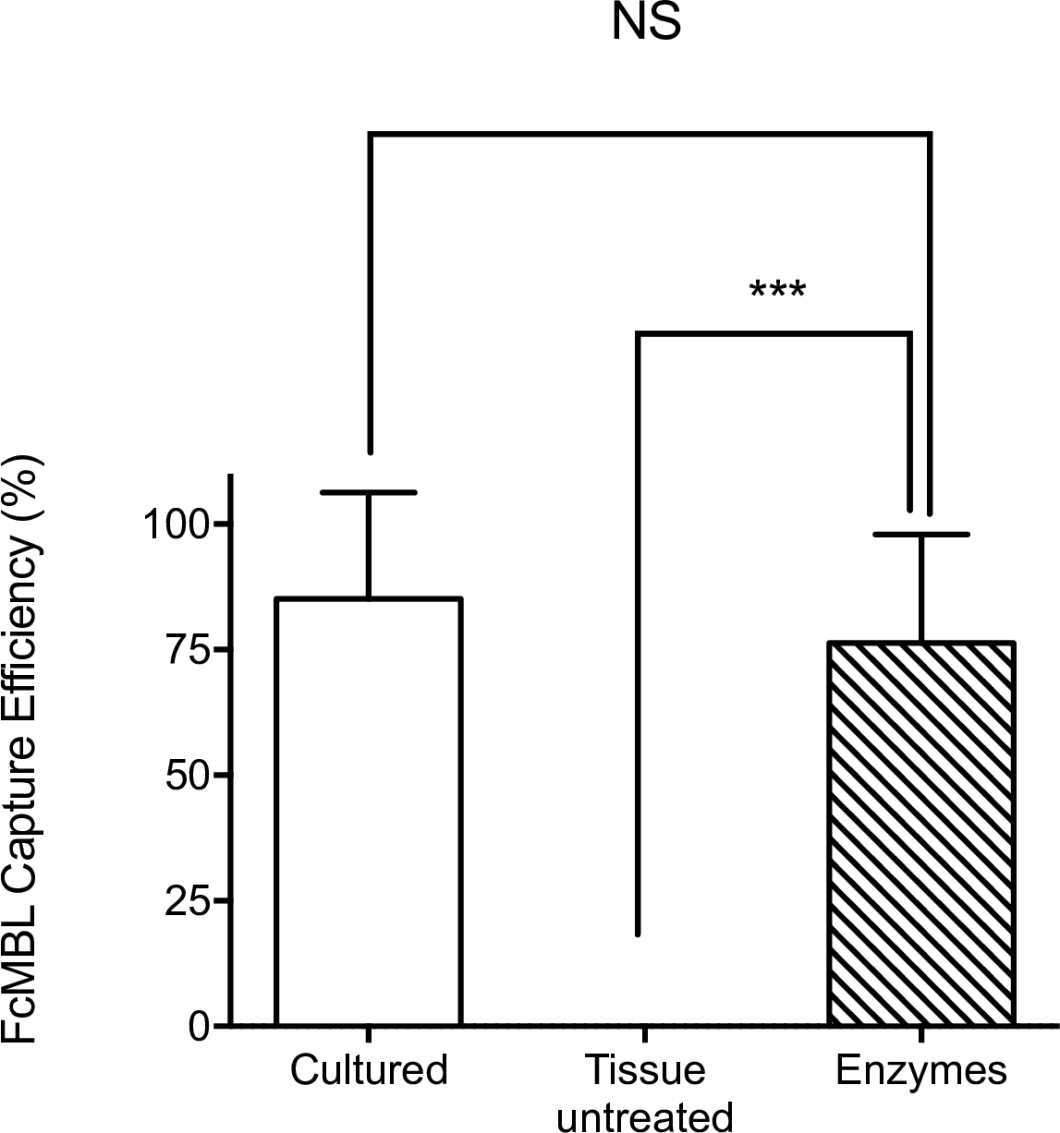
Restoration of FcMBL-mediated capture of *S*. *aureus* from infected articular fluids and synovial tissues. The high level (mean: 85% +/- 5.9) of FcMBL-mediated capture of cultured *S*. *aureus* strains (Cultured) was greatly reduced (mean: 1.3% +/- 1.2) when similar studies were carried out by adding FcMBL-beads directly to complex tissue samples. Addition of an enzymatic cocktail of proteases and hyaluronidase (Enzymes) to remove endogenous Ig and other potential surface masking proteins, as well as decrease sample viscosity, resulted in restoration of FcMBL binding (mean: 76% +/- 5.7). ***: p-value < 0.0001.

Another potential factor that could contribute to this variability is the high viscosity of articular fluids, which is due in part to the presence of large natural carbohydrate polymers (glycosaminoglycans), such as hyaluronic acid [19]. When we pre-treated the synovial fluid samples with hyaluronidase, we were able to homogenize the samples and results became more consistent (Fig 5). More importantly, by treating the pellet with a mixture of proteases and hyaluronidase, we were able to almost fully restore FcMBL’s ability to bind to the bacteria, resulting in capture almost all of the pathogens present in clinical tissue samples, with a mean capture efficiency of 76%, which is close to that observed using cultured *S*. *aureus* isolates (Fig 5). Similar FcMBL capture results were obtained with various types of clinical specimens, Table 1.

As a proof-of-concept, to confirm beads capture maintain diagnostic potential, we performed downstream laboratory assays commonly used in state of the art clinical microbiology laboratories on articular fluids stored at 4°C for less than one week. We analyzed the bead-processed samples on a Genexpert platform for RT-PCR analysis and on a Bruker Daltonics MALDI-TOF MS Biotyper identification platform. For RT-PCR, we had no invalid results due to inhibitors or viscous samples following capture on FcMBL magnetic beads, which allowed us to concentrate pathogens from large volumes (up to 5 ml samples), which enhanced the sensitivity of the test (Table 2). Rapid identification was attempted using MALDI-TOF after one to three hours of culture of beads using sample and bead amounts identical to those tested by RT-PCR. No peaks were generated after one or two hours culture but scores greater than 2 were generated after three hours of growth, allowing species level identification of *S*. *aureus* using MALDI-TOF.

**Table 2:** PCR results with articular fluids infected with *S*. *aureus*.

Patient	Bacterial Load CFU/ml	Without capture^a^ Ct spa^b^	Vol. used after FcMBL capture	Ct spa after FcMBL capture
A	1.10^7^	Invalid	100 μl	21
B	8.10^4^	22	100 μl	24
C	1.10^4^	30	100 μl	31
D	1.10^4^	Invalid	100 μl	32
E	1.10^3^	34	1 ml	31
F	2.10^2^	37	1 ml	31
G1	1.10^2^	Negative	100 μl	Negative
G2	1.10^2^	Negative	5 ml	32
H	3.10^1^	Negative	1 ml	34

^a^ Without capture, the volume defined for PCR is 100 μl as per manufacturer’s instructions.

^b^ Number of amplification cycles required to detect staphylococcal protein A (spa) gene as reported on the Genexpert software.

## Discussion

The main goal of this study was to determine whether magnetic beads coated with the generic pathogen capture protein, FcMBL, could be used to isolate *S*. *aureus* pathogens from infected tissues of osteoarthritis patients and recover enough bacteria to bypass the conventional culture step. We initially confirmed that FcMBL beads provide an efficient method to capture *S*. *aureus* strains that were obtained as isolates from our clinical tissue samples and cultured prior to analysis; however, we could not capture these bacteria when FcMBL-beads were added directly to the complex joint fluids or infected tissue samples. Analysis of the mechanism revealed that it was largely due to the presence of high concentrations of proteins in the infected joint samples, such as Ig, which coat the surface of the bacteria and mask FcMBL’s cell surface binding sites (the external cell wall carbohydrate WTA [20] and protein A [18]). Interestingly, anti-WTA IgG is often found in the serum of patients infected with *S*. *aureus*, and it is a trigger of complement-mediated opsonophagocytosis that activates the classical complement pathway [21]. MBL binding of pathogens also has been shown to be lower in adults than in infants because of the presence of higher levels of inhibitory anti-WTA antibodies [22]. It is likely for this reason that removing the supernatant (and Ig proteins contained within it) from cell pellets increased FcMBL capture of bacteria in the present study. Finally, the addition of hyaluronidase to decrease viscosity, and a protease mixture to remove any remaining surface bound Ig as well as other bound endogenous proteins that could inhibit FcMBL binding (e.g., bound native MBL, C-reactive protein, etc.), increased the efficiency of FcMBL capture even further, making it useful for direct isolation of live *S*. *aureus* pathogens from clinical samples.

The cell surface determinants that are the ligands of the carbohydrate recognition domain of FcMBL are highly conserved and hence, they do not exhibit the variability that can be observed with targets for specific antibodies [18]. While the Fc portion of the FcMBL was also shown to mediate capture of cultured *S*. *aureus* strains on its own by binding to endogenous protein A on the surface of these bacteria, the clinical samples of *S*. *aureus* exhibited much greater heterogeneity of binding. As bacterial cell surface protein A binds the Fc portion of endogenous Ig (as well as FcMBL), some of this heterogeneity might be due to bound Ig interfering with Fc bead binding. However, even when the cells were treated with the protease mixture to remove Ig, the Fc-beads were not nearly as effective as the FcMBL beads for pathogen capture using clinical samples. This is likely because protease digestion removes surface glycoprotein receptors for cells, complement, and antibodies, including protein A. The use of Fc beads also can be limited by variation in the expression of protein A variants, which can differ depending on the bacterial strain, type of infection, and culture condition [23, 24]. In contrast, the protease treatments do not alter surface carbohydrates, and thus, they did not interfere with FcMBL binding to these cells.

Protein concentrations in articular fluids range from 20 to 40 g/l, and Igs, mostly IgGs, represent half of these proteins [19]. The broad-range action of the proteases we use to treat our clinical samples also degrades other proteins found in biological fluids, such as endogenous MBL, complement and cytokines, which bind to the bacteria and thus, could contribute to masking of FcMBL binding. Hyaluronidase, the other enzyme we tested, degrades hyaluronic acid, which is one of the major lubricants of the articular joints. It is also largely responsible for the high viscosity of joint fluids, making them difficult to handle for clinical analysis, and for this reason, hyaluronidase treatments are routinely used to improve analysis measurements [25]. In addition, as hyaluronic acid is a complex carbohydrate polymer, it also could interfere with binding of FcMBL to *S*. *aureus*. Indeed, we found that adding hyaluronidase reduced fluid viscosity and enabled us to produce more consistent and efficient pathogen capture using the FcMBL beads. A potential limitation of this study is that it was designed as a retrospective study with frozen clinical samples. But we were able to culture the expected live microorganisms from 100% of the samples, and we obtained similar capture results with samples that were stored in the cold for less than a week in our PCR studies.

While we show that *S*. *aureus* can be extracted from challenging clinical samples, MBL has been reported to bind over 90 different microbial species [12] that can be relevant in patient care. The ability of FcMBL beads combined with the protease treatment to perform similarly with other species needs to be further evaluated in clinical samples. Moreover the capture of bacteria in polymicrobial samples is highly variable depending on the relative concentrations of microorganisms of each species, and on the species of these microorganisms. Numerous polymicrobial samples will have to be tested to determine how it performs in real-life conditions.

In summary, the FcMBL magnetic bead capture technology provides a simple and rapid process to extract *S*. *aureus* capable of growth from patient tissue samples, which potentially could be used in clinical laboratories to bypass the day to multi-day long culture step that is currently required for pathogen identification and AST. The method still needs to be optimized for laboratory workflow and patient care needs, and then evaluated in a future prospective clinical study before its clinical relevance can be fully assessed.
